# SEEG in 3D: Interictal Source Localization From Intracerebral Recordings

**DOI:** 10.3389/fneur.2022.782880

**Published:** 2022-02-08

**Authors:** David Satzer, Yasar T. Esengul, Peter C. Warnke, Naoum P. Issa, Douglas R. Nordli

**Affiliations:** ^1^Department of Neurosurgery, University of Chicago, Chicago, IL, United States; ^2^Department of Neurology, University of Chicago, Chicago, IL, United States; ^3^Section of Child Neurology, Department of Pediatrics, University of Chicago, Chicago, IL, United States

**Keywords:** electrical source localization, epilepsy, inverse problem, irritative zone, stereotaxy

## Abstract

**Background:**

Stereo-electroencephalography (SEEG) uses a three-dimensional configuration of depth electrodes to localize epileptiform activity, but traditional analysis of SEEG is spatially restricted to the point locations of the electrode contacts. Interpolation of brain activity between contacts might allow for three-dimensional representation of epileptiform activity and avoid pitfalls of SEEG interpretation.

**Objective:**

The goal of this study was to validate SEEG-based interictal source localization and assess the ability of this technique to monitor far-field activity in non-implanted brain regions.

**Methods:**

Interictal epileptiform discharges were identified on SEEG in 26 patients who underwent resection, ablation, or disconnection of the suspected epileptogenic zone. Dipoles without (free) and with (scan) gray matter restriction, and current density (sLORETA and SWARM methods), were calculated using a finite element head model. Source localization results were compared to the conventional irritative zone (IZ) and the surgical treatment volumes (TV) of seizure-free vs. non-seizure-free patients.

**Results:**

The median distance from dipole solutions to the nearest contact in the conventional IZ was 7 mm (interquartile range 4–15 mm for free dipoles and 4–14 mm for scan dipoles). The IZ modeled with SWARM predicted contacts within the conventional IZ with 83% (75–100%) sensitivity and 94% (88–100%) specificity. The proportion of current within the TV was greater in seizure-free patients (*P* = 0.04) and predicted surgical outcome with 45% sensitivity and 93% specificity. Dipole solutions and sLORETA results did not correlate with seizure outcome. Addition of scalp EEG led to more superficial modeled sources (*P* = 0.03) and negated the ability to predict seizure outcome (*P* = 0.23). Removal of near-field data from contacts within the TV resulted in smearing of the current distribution (*P* = 0.007) and precluded prediction of seizure freedom (*P* = 0.20).

**Conclusions:**

Source localization accurately represented interictal discharges from SEEG. The proportion of current within the TV distinguished between seizure-free and non-seizure-free patients when near-field recordings were obtained from the surgical target. The high prevalence of deep sources in this cohort likely obscured any benefit of concurrent scalp EEG. SEEG-based interictal source localization is useful in illustrating and corroborating the epileptogenic zone. Additional techniques are needed to localize far-field epileptiform activity from non-implanted brain regions.

## Introduction

For epilepsy surgery to render a patient seizure-free, the epileptogenic zone must be accurately localized. Stereo-electroencephalography (SEEG) obtained from multi-contact depth electrodes can help to define the epileptogenic zone. Electrode placement is dictated by the pre-implantation hypothesis, which is based on non-invasive data including semiology, scalp EEG, and radiographic abnormalities (1).

Conventional SEEG analysis is prone to the streetlight effect, in which a search process is restricted to illuminated places (i.e., the depth electrode contact locations). If the pre-implantation hypothesis is incorrect and no depth electrode contacts are located within the epileptogenic zone, the earliest observed epileptiform activity reflects propagation. Failure to recognize propagated activity as such may preclude postoperative seizure freedom (2).

Despite the three-dimensional configuration of depth electrodes, interpolation of electrical activity within non-implanted brain regions is not feasible without sophisticated modeling. This problem has been addressed for scalp EEG using electrical source localization. Source localization attempts to solve the “inverse problem”, in which electrode locations and potentials are known, and the intracranial electrical sources are unknown (3). The ability to use SEEG for source localization was recently introduced in a commercial software package but has not yet been validated.

Most published research on source localization with SEEG comes from computational simulations. Multiple simulation studies found that under ideal circumstances, sources within 1.5–3 cm of the nearest electrode contact can be localized with an error of <1 cm (4, 5). One study found superior localization accuracy with combined scalp EEG and SEEG compared to scalp EEG or SEEG alone (6). An analysis of real SEEG recordings found 95% concordance between the clinically defined epileptogenic zone and an automated contact-ranking method based on ictal low frequency suppression and multi-band fast activity (7). Source localization of subdural recordings with and without SEEG has been found to be superior to conventional interpretation of intracranial EEG (8).

Source localization might facilitate three-dimensional interpretation of SEEG, as the prefix “stereo” implies SEEG is meant to be. Moreover, in patients with an incorrect pre-implantation hypothesis, source localization might allow accurate interpretation of SEEG data and optimize postoperative seizure outcomes for patients with difficult-to-localize epilepsy. The development of a commercially available platform has made this technique readily accessible. Therefore, the goal of the present study is to optimize and validate SEEG-based source localization of the irritative zone (IZ) based on conventional SEEG interpretation and the surgical treatment volumes (TV) of seizure-free and non-seizure-free patients.

## Methods

### Protocol

This study was approved by the University of Chicago institutional review board (protocol IRB20-1668). Informed consent was waived for this retrospective study. Patients who underwent implantation of multiple depth electrodes (>15 contacts) and subsequent resection, ablation, and/or disconnection of the suspected epileptogenic zone from 2014–2020 were included. Patients with multifocal ictal onset were excluded. A minimum of 1 year of follow-up after the most recent surgery was required.

### Surgical Procedure

Patients underwent SEEG evaluation to characterize the epileptogenic zone in the setting of discordant or inconclusive non-invasive data (e.g., bilateral interictal activity, no radiographic lesion, or unclear extent of epileptogenic zone). Depth electrodes were placed using a frame-based technique (CRW, Integra Neurosciences, Plainsboro, NJ, USA). When used, subdural strip electrodes were placed after depth electrodes *via* a separate burr hole. All electrode implantations were performed by the same neurosurgeon (PCW).

### Data Acquisition

SEEG was recorded at a sampling rate of 1,024 Hz using an XLTEK amplifier system (Natus Medical Incorporated, Pleasanton, California, USA). In most cases, concurrent scalp EEG was recorded using a complete or partial 10–20 configuration. SEEG and scalp EEG were recorded in reference to FCz. Antiseizure medications were usually reduced or held during the monitoring to facilitate the recording of seizures. Seizure outcomes were assessed during outpatient visits or phone interviews. Engel class at last follow-up was recorded.

### EEG Processing

Source localization was performed using Curry eight (Compumedics Neuroscan, Hamburg, Germany). Representative SEEG recordings containing at least 25 interictal epileptic discharges (IEDs) were analyzed, along with concurrent scalp EEG (if obtained). Recordings from subdural electrodes (if present) were not analyzed, since these were obtained in few cases and greatly differed from scalp EEG given their high signal-to-noise (SNR) ratio and from SEEG given their location outside the TV by virtue of their extra-axial location. Bad electrodes [channels with non-reducible artifact, channels with prominent 60 Hz artifact indicating aberrant impedance, and depth electrode contacts located outside the dura or in cerebrospinal fluid (CSF)] were removed from analysis. EEG was filtered with a Hann fast Fourier transform from 1–100 Hz and a 60 Hz notch filter.

Monomorphic IEDs were manually identified by one author (YTE) and verified by a board-certified pediatric epileptologist (DRN), both of whom were blind to clinical outcomes. When multifocal IEDs were seen, only the subgroup of IEDs involving similar channels as ictal onset was used. Each contact was classified as being inside or outside the conventional IZ based on the presence or absence of visible IEDs; indeterminate contacts were not classified. IEDs were then averaged, aligned to the SEEG channel in which the IED first appeared; a common average reference was applied automatically after averaging. The decision to average IEDs was based on associated activity (e.g., slow wave discharges) as well as the common average re-referencing mandated by the software. The window from 500–1000 ms prior to IED peak was used for noise estimation. Source localization was performed at the half-rise point, defined as the midpoint between the start and peak of the dominant deflection within the averaged IED from SEEG.

### Model Generation

Preoperative gadolinium contrast-enhanced T1-weighted MRI, CT after depth electrode placement, and post-treatment (resection, ablation, or disconnection) MRI were coregistered using Curry.

Electrode locations were determined from CT. The TV was defined based on MRI from postoperative day one in patients who underwent resection or disconnection, and immediate post-ablation MRI in patients who underwent laser interstitial thermal therapy (LITT). For LITT, the TV was defined as the contrast-enhancing volume including the hypointense center. For patients that underwent multiple epilepsy surgeries after SEEG, the analyzed TV was the union of the TVs from all post-SEEG surgeries.

Gray matter was segmented on preoperative MRI using SPM12 (fil.ion.ucl.ac.uk/spm). Potential source locations for current density and scanning dipole modeling were defined by a grid of points, without prespecified orientations, distributed every 2 mm throughout the gray matter volume.

Two finite element models were generated. A three-compartment model (capable of modeling scalp and intracranial recordings) contained representations of the scalp, skull, and intracranial space (with respective conductivity of 0.33, 0.0042, and 0.33 S/m). A 1-compartment model (capable of modeling intracranial recordings only) included only the intracranial space. A 2-mm mesh was created, with refinement (1.4-mm mesh) near electrode contacts.

### Source Localization

To determine the optimal SEEG source localization method, multiple methods were applied to SEEG without scalp EEG. Two single-source equivalent current dipoles were fit using previously reported methods (9, 10): a free dipole constrained to the intracranial space, and a scan dipole constrained to the previously defined grid of gray matter points. Current density reconstruction was instituted using standardized low resolution tomographic analysis (sLORETA), which yields an F-statistic for each source location (11), and sLORETA-weighted accurate minimum norm (SWARM), which gives current values for each source location (12). Several percent-maximum lower cutoff values (10, 50, 75, 90, 95, and 99%) were applied to current density results, so that only values (F-statistics or current densities) greater than that percentage of the maximum point value were included.

Concordance between source localization results and the conventional IZ was assessed first. This analysis was restricted to seizure-free patients, in whom the epileptogenic zone was known to be sampled. The distance from each dipole to the nearest contact showing interictal activity was calculated. Current density was used to predict interictal activity in contacts within 10 mm of a gray matter point with above-cutoff current density, vs. contacts farther removed from the modeled IZ. This estimated sampling distance of 10 mm was based on precision data from modeling studies (4). The optimal cutoff values for sLORETA and SWARM were based on maximum value of Youden's J statistic (sensitivity + specificity - 1).

Congruence between source localization results and the TV was compared between seizure-free (Engel class I outcome at last follow-up) and non-seizure-free groups. The shortest distance between each dipole and the TV boundary/interior was measured. For sLORETA, the percentage of above-cutoff gray matter points within the TV was determined. For SWARM, the percentage of summed current within the TV was calculated. For both current density methods, the TV was dilated by 2 mm to account for variability introduced by the grid spacing.

Further analysis was performed by including scalp EEG data or by excluding depth electrode contacts within 2 mm of the TV. When scalp EEG was included, a 3-compartment head model was used, and common average referencing was applied separately to SEEG and scalp EEG.

### Statistical Analysis

Data were compared with the Mann-Whitney U test if unpaired and the Wilcoxon sign-rank test if paired. An alpha level of 0.05 was used for all significance testing. Statistical analysis was performed with SAS OnDemand for Academics (version 9.4, SAS Institute Inc., Cary, NC, USA). Unless stated otherwise, results are reported as either mean ± standard deviation or median [interquartile range (IQR)].

## Results

### Cohort

Records from 49 patients who underwent SEEG were reviewed. Patients were excluded due to no resection, ablation, or disconnection surgery after SEEG (10 patients), unavailable SEEG recordings (six patients), unavailable postoperative imaging (two patients), <1 year of follow up (one patient), SEEG-proven bilateral mesial temporal lobe epilepsy (two patients), insufficient (15 or fewer) intraparenchymal contacts (one patient), and no IEDs visible on SEEG (one patient).

Twenty-six patients met inclusion criteria (Table 1). Age was 29 ± 13 years at the time of SEEG. Ten patients (38%) were male, and five patients (19%) had epilepsy surgery prior to SEEG investigation. Ten patients (38%) had a single lesion visible on MRI, three patients (12%, all of whom had tuberous sclerosis) had multiple lesions, and 13 patients (50%) had a normal brain MRI. The solitary lesions included mesial temporal sclerosis (five patients), focal cortical dysplasia (two patients), cerebral cavernous malformation (one patient), pilocytic astrocytoma (one patient), and pleomorphic xanthoastrocytoma (one patient). Nineteen (73%) patients were determined to have temporal lobe epilepsy based upon non-invasive and SEEG evaluation.

**Table 1:** Patient demographics.

	**Full cohort**		**Engel I**	
**Demographic**	***N***	**% of cohort**	***N***	**% Engel I**
**Total**	26	100%	11	42%
**Sex**				
Male	10	38%	3	30%
Female	16	62%	8	50%
**Prior surgery**				
Yes	5	19%	2	40%
No	21	81%	9	43%
**Radiographic lesion**				
Single	10	38%	5	50%
Multiple	3	12%	2	67%
None	13	50%	4	31%
**Consensus localization**				
Temporal	19	73%	9	47%
Temporal mesial	15	58%	8	53%
Temporal neocortical	4	15%	1	25%
Frontal	2	8%	0	0%
Insular/opercular	2	8%	1	50%
Parietal	1	4%	0	0%
Multilobar	2	8%	1	50%
**Epilepsy surgery**				
LITT (single)	17	65%	5	29%
LITT (multiple)	6	23%	4	67%
Resection	1	4%	0	0%
LITT followed by resection	1	4%	1	100%
LITT followed by disconnection	1	4%	1	100%

*LITT, laser interstitial thermal therapy*.

Ablation *via* laser interstitial thermal therapy (LITT) was performed after SEEG in all but one patient. Initial resection, delayed resection, and delayed posterior quadrant disconnection were each performed in one patient. Eight patients (31%) underwent multiple epilepsy surgeries after SEEG. Follow-up was 2.8 ± 1.7 years after the most recent epilepsy surgery. Eleven patients (42%) were seizure-free (Engel class I) at last follow-up.

### Concordance With the Conventional IZ

**Figure 1:**
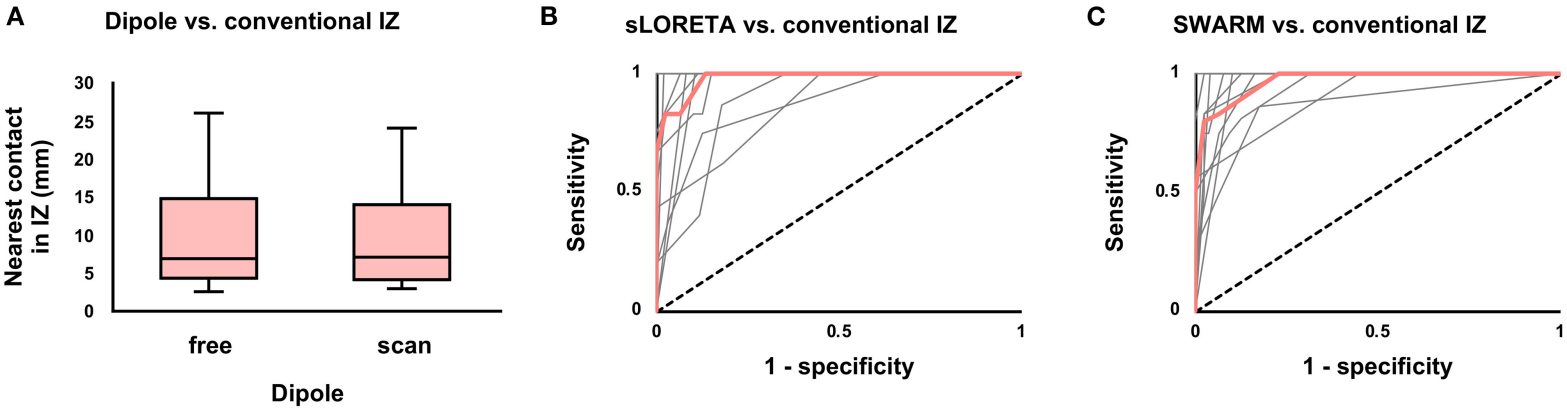
Agreement between the conventional and modeled irritative zones (IZ) among seizure-free patients. **(A)** Boxplot of distance from dipole to nearest contact showing interictal activity. There was no difference (*P* = 0.66) between free and scan dipole solutions. **(B)** Receiver-operating characteristic (ROC) curve for concordance of the conventional IZ with the sLORETA-modeled IZ (volume of above-cutoff current density). Median (thick red line) and individual patient curves (thin gray lines) are shown. **(C)** Similar ROC curve for SWARM.

SEEG-based source localization solutions were first compared to the conventional IZ (based on visual review of EEG) in patients with Engel I outcomes. Median distance from dipole to the nearest contact showing interictal activity was 7 mm (4–15 mm) for free dipole solutions and 7 mm (4–14 mm) for scan dipole solutions (Figure 1A). Because the accuracy of the two dipole models did not differ (*P* = 0.66), the free dipole model was used to attempt to predict seizure outcome given its lower computational demand.

Current density was used to predict interictal activity in contacts within a 10 mm sampling distance of the modeled IZ. For sLORETA, Youden's J statistic was greatest at the 75% cutoff, at which point sensitivity was 100% (87–100%) and specificity was 87% (82–94%). For SWARM, Youden's J statistic was greatest at the 50% cutoff, at which point sensitivity was 83% (75–100%) and specificity was 94% (88–100%). Full test statistics are listed in Table 2. Receiver-operating characteristic (ROC) area under the curve (AUC) was 97% (88–99%) for sLORETA and 97% (94–98%) for SWARM (Figures 1B,C). AUC did not differ (*P* = 0.73) between sLORETA and SWARM, and so sLORETA (with a 75% cutoff) and SWARM (with a 50% cutoff) were both subsequently used to attempt to predict seizure outcome.

**Table 2:** Test statistics for agreement of modeled irritative zone with conventional irritative zone of seizure-free patients and with treatment volume of seizure-free vs. non-seizure-free patients.

**Ground truth**	**Conventional IZ^a^**		**TV + outcome**
**Modeled IZ**	**sLORETA**	**SWARM**	**SWARM**
Sensitivity	100% (87–100%)	83% (75–100%)	45%
Specificity	87% (82–94%)	94% (88–100%)	93%
PPV	59% (36–80%)	76% (50–100%)	83%
NPV	100% (88–100%)	98% (90–100%)	70%
Accuracy	87% (83–94%)	89% (85–96%)	73%
Youden's J	85% (69–94%)	81% (69–88%)	39%
AUC	97% (88–99%)	97% (94–98%)	70%

*IZ, irritative zone; TV, treatment volume; PPV, positive predictive value; NPV, negative predictive value; AUC, area under the curve*.

a *Median (interquartile range)*.

### Prediction of Outcome With SEEG Only

For outcome-based assessment of SEEG-based source localization, SEEG without scalp EEG was analyzed. The median number of depth electrodes was six (IQR 5–7), with 46 (40–53) contacts located within the brain. After removal of bad channels, 44 (38–51) contacts remained.

The distance between free dipole solutions and the TV did not differ between seizure-free (median 9 mm, IQR 0–9 mm) and non-seizure-free (median 5 mm, IQR 3–22 mm) patients (*P* = 0.79). For sLORETA, the percentage of above-cutoff gray matter points within the TV did not vary (*P* = 0.53) by seizure outcome.

**Figure 2:**
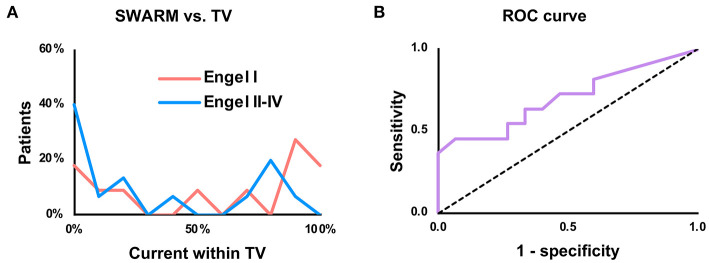
Prediction of seizure freedom based upon proportion of current within the treatment volume (TV). **(A)** Histogram comparing proportion of above-cutoff current within the TV, using a lower cutoff of 50% of the maximum point current. “Patients” refers to proportion of patients in each outcome group (Engel I vs. Engel II–IV) within each histogram bin. **(B)** Receiver-operating characteristic (ROC) curve for SWARM with a 50% cutoff.

For SWARM, the proportion of current within the TV was greater (*P* = 0.04) in seizure-free patients (median 71%, IQR 13–93%) than non-seizure-free patients (median 16%, IQR 0–75%; Figure 2A). Youden's J statistic was greatest using an 80% within-TV threshold, at which point sensitivity was 45% and specificity was 93%; full test statistics are listed in Table 2. ROC AUC was 70% (Figure 2B).

**Figure 3:**
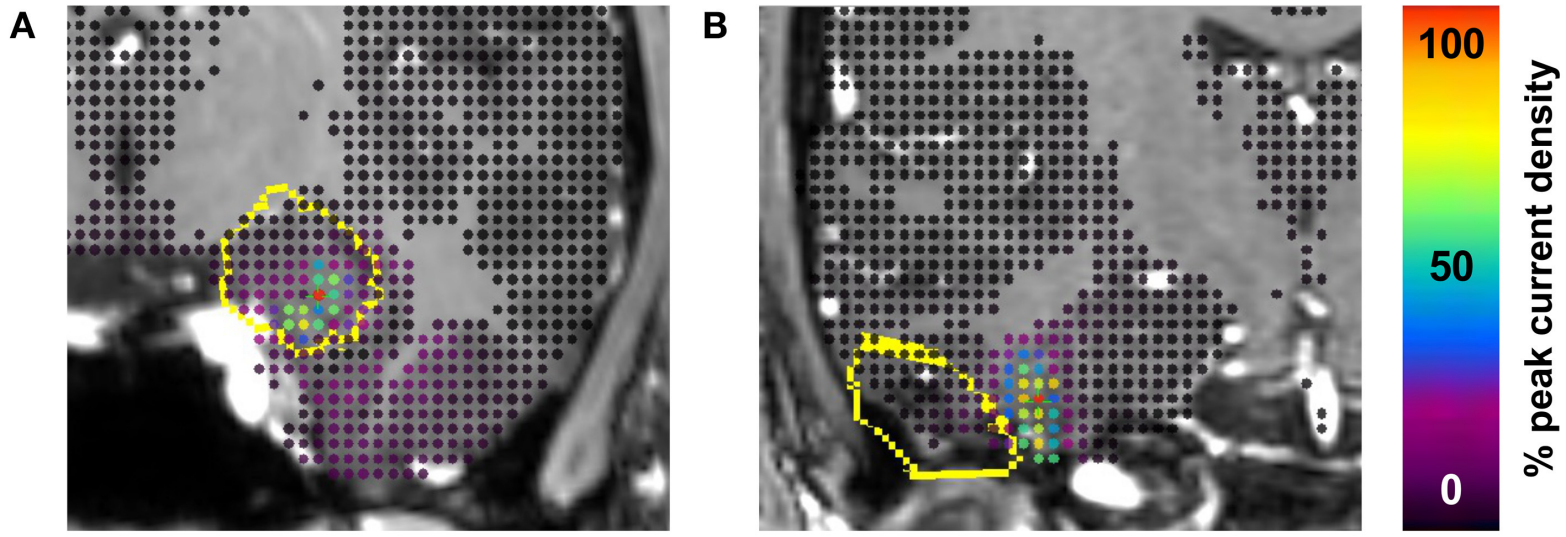
Examples of true positive and true negative cases. **(A)** Engel class I outcome 2 years after laser ablation of amygdala and hippocampus including 92% of interictal current. **(B)** Engel class III outcome 2 years after resection of temporal pilocytic astrocytoma including 0% of interictal current. The treatment volume (dilated by 2 mm to account for grid size) is outlined in yellow on coronal section from preoperative MRI. Current density is shown at each point in the gray matter grid. Proportions of current included in the treatment volume are based on the SWARM method with a lower cutoff value of 50% of the maximum point current.

Examples of true positive (seizure-free with high proportion of current within the TV) and true negative (non-seizure-free with low proportion of current within the TV) cases are illustrated in Figure 3. Based on the ability to predict seizure freedom from SEEG recordings, SWARM was used for all subsequent analyses.

### Effects of Including Scalp EEG

**Figure 4:**
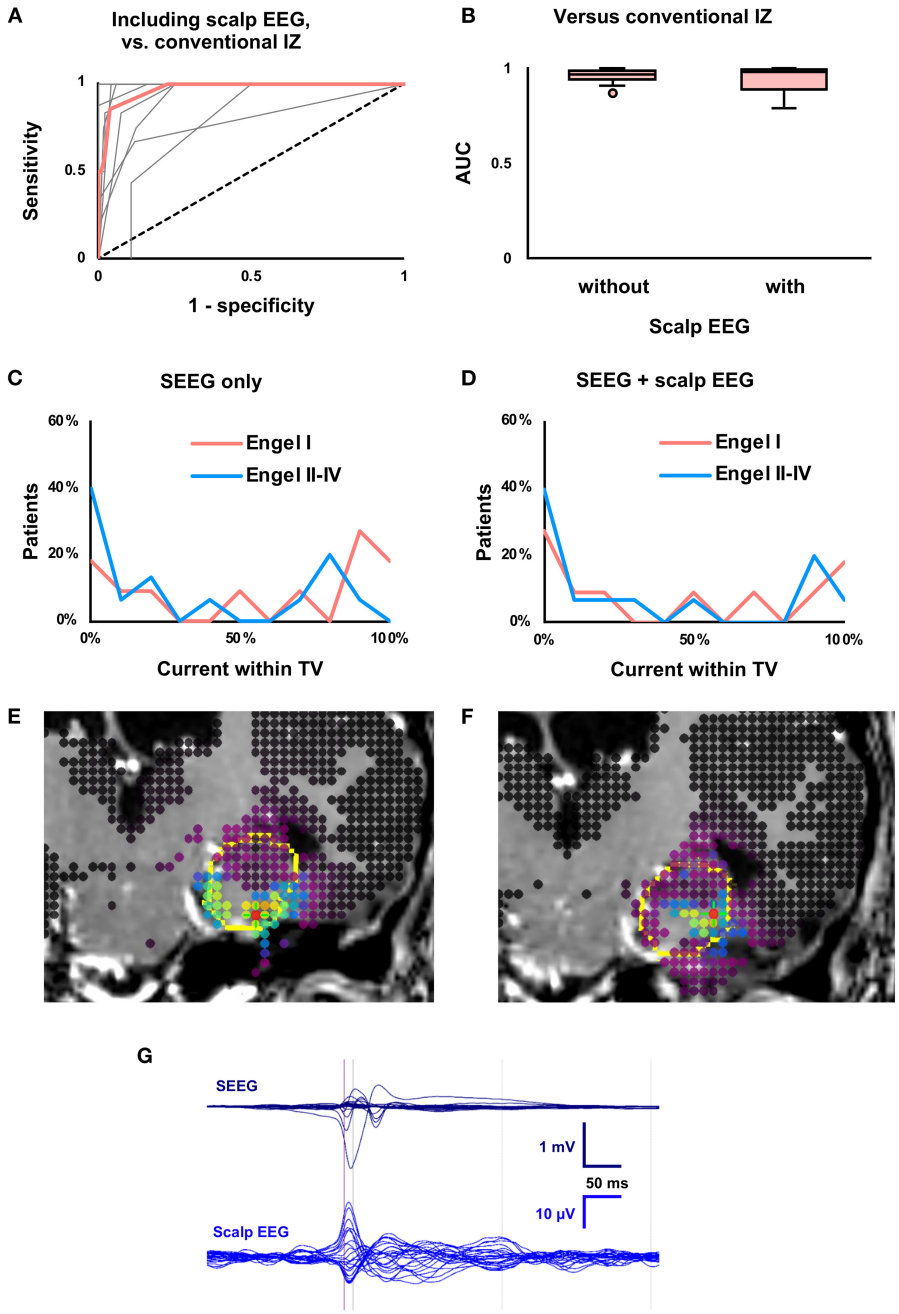
Effects of including concurrent scalp EEG. **(A)** Receiver-operating characteristic (ROC) curve for concordance of the conventional and modeled irritative zones (IZ) when scalp EEG was included. Median (thick red line) and individual patient curves (thin gray lines) are shown. **(B)** Boxplot of ROC area under the curve (AUC), which was not affected (*P* = 0.38) by inclusion of scalp EEG. **(C)** Using SEEG, the proportion of current within the treatment volume (TV) predicted seizure outcome, as in Figure 2A. **(D)** Percent overlap of the modeled IZ with the TV was unable to predict seizure outcome (*P* = 0.23) when concurrent scalp EEG was also analyzed. **(E)** SEEG-derived current density map (as in Figure 3) from patient with previously resected temporal pleomorphic xanthoastrocytoma with Engel I outcome after laser ablation of amygdala and hippocampus. **(F)** Current density map derived from both SEEG and scalp EEG in the same patient. Note the lateral shift in the modeled IZ. **(G)** Butterfly plot of averaged interictal epileptiform discharges on SEEG and concurrent scalp EEG from the same patient. The time point used for source localization (half-rise point in SEEG) is indicated by the purple vertical line.

Twenty-six (13, 14) scalp electrodes were placed, and 26 (13–15) remained after removal of bad channels. Two patients did not undergo concurrent scalp EEG. When the modeled IZ and the conventional IZ were compared, ROC AUC did not vary (*P* = 0.38) based on the inclusion of scalp EEG (Figures 4A,B). As in the SEEG-only condition, Youden's J statistic was greatest at the 50% cutoff. When scalp EEG was included, the percent of current within the TV did not vary (*P* = 0.23) between seizure-free (median 36%, IQR 6–86%) and non-seizure-free (median 14%, IQR 0–77%) patients (Figures 4C,D).

Among seizure-free patients, including scalp EEG led to a 0.3 (0.1–0.7) cc smaller (*P* = 0.048) modeled IZ. The distance between the maximum point current and the nearest scalp electrode was two (0–5) mm shorter (*P* = 0.03) with scalp EEG (median 47, IQR 36–52 mm) than without scalp EEG (median 51 mm, IQR 43–53 mm). Figures 4E–G show a representative case with a more superficial modeled IZ when scalp EEG was included. In the full cohort, the size of the modeled IZ and the distance from peak current to nearest scalp electrode did not vary by inclusion of scalp EEG.

### Prediction of Far-Field Activity

**Figure 5:**
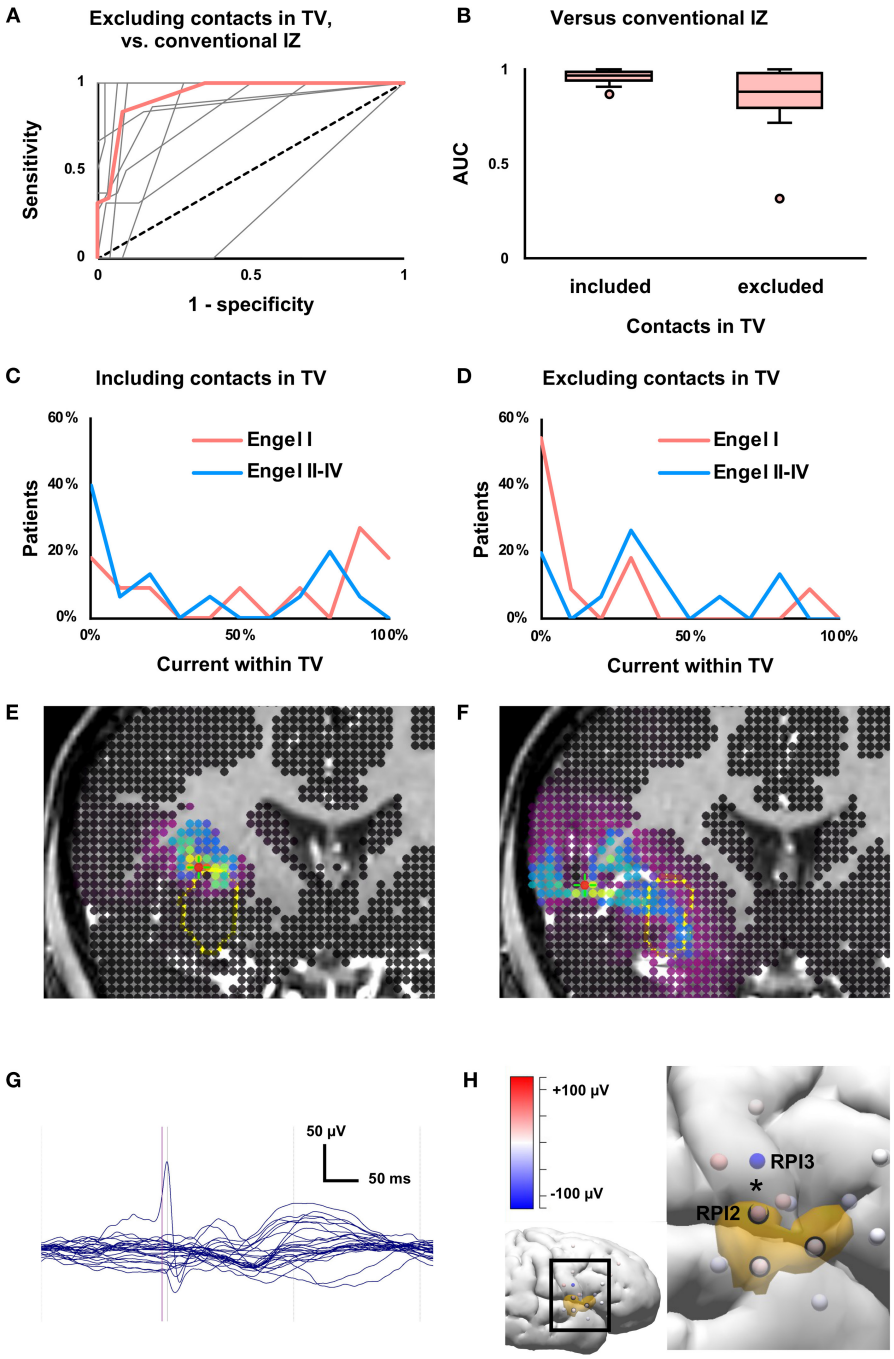
Effects of excluding near-field data from contacts within treatment volume (TV). **(A)** Receiver-operating characteristic (ROC) curve for concordance of the conventional and modeled irritative zones (IZ) when contacts within the TV were excluded from analysis. Median (thick red line) and individual patient curves (thin gray lines) are shown. **(B)** Boxplot of ROC area under the curve (AUC), which was not significantly affected (*P* = 0.05) by exclusion of contacts within the TV. **(C)** The proportion of current within the treatment volume (TV) predicted seizure outcome (as in Figure 2A), but **(D)** not when within-TV contacts were excluded (*P* = 0.22). **(E)** Current density map (as in Figure 3) derived from all depth electrode contacts, from patient with Engel I outcome after laser ablation of insula. In this case, the modeled IZ is located along the border of the TV. **(F)** Exclusion of contacts within the TV resulted in smearing of current density. **(G)** Butterfly plot of averaged interictal epileptiform discharges on SEEG from the same patient, showing few channels with high-amplitude discharges. **(H)** Three-dimensional depiction of relationship between depth electrode contacts (spheres) and TV (orange volume). Inset shows the brain region of interest. Contacts within the TV are circled in black. Contacts are colored according to voltage at the half-rise point. Removing contacts within the TV led to the loss of a high-amplitude phase reversal (*) between adjacent insular contacts RPI2 and RPI3.

To assess the ability of SEEG-based source localization to predict far-field activity, contacts within 2 mm of the TV were excluded. On average, six (3–12) contacts were excluded, and 40 (32–45) contacts remained. Three patients had 15 or fewer contacts outside the TV and were excluded from this analysis. When the modeled IZ and the conventional IZ were compared, there was a non-significant (*P* = 0.05) decrease in ROC AUC when contacts within the TV were excluded (median 88%, IQR 79–97%) compared to when those contacts were included (median 97%, IQR 94–98%; Figures 5A,B). As in the SEEG-only condition, Youden's J statistic was greatest at the 50% cutoff. When recordings from contacts within the TV were excluded, the percent of current within the TV did not vary (*P* = 0.22) between seizure-free (median 2%, IQR 0–24%) and non-seizure-free (median 31%, IQR 17–36%) patients (Figures 5C,D).

Among seizure-free patients, the proportion of current within the TV was 20% (1–67%) smaller (*P* = 0.04) when contacts within the TV were excluded. In the full cohort, removing contacts in the TV led to a 0.3 (0–1.1) cc larger (*P* = 0.007) modeled IZ. Removing within-TV contacts did not affect the fraction of current within the TV in the full cohort (*P* = 0.13) or the size of the modeled IZ in seizure-free patients (*P* = 0.16).

Figure 5E shows a representative case with a modeled IZ partially overlapping the TV. When contacts within the TV were excluded, the modeled IZ was smeared, and minimally overlapped the TV (Figure 5F). On SEEG, high-amplitude IEDs were seen in few channels, and removing some of these channels led to the loss of local signals (e.g., a high-amplitude phase reversal between adjacent contacts on the same depth electrode) with preservation of volume-conducted potentials (Figures 5G,H).

## Discussion

The goals of this study were to optimize and validate SEEG-based interictal source localization and to explore whether the technique could be used to model far-field activity in non-implanted brain regions. The median distance from dipole solutions to the nearest contact with visible IEDs was 7 mm, and the IZ modeled using SWARM agreed with the conventional IZ with 83% sensitivity and 94% specificity. The proportion of interictal current contained within the TV predicted seizure outcome with 45% sensitivity and 93% specificity.

To simulate the situation in which no electrode contacts are located in the epileptogenic zone, source localization was repeated omitting SEEG data from contacts within the TV, which in seizure-free patients must contain the epileptogenic zone (16). In the absence of near-field data from contacts within the TV, SEEG-based interictal source localization could not distinguish seizure-free and non-seizure-free patients.

### Validation of Scalp EEG-Based Source Localization

The present study drew its design from previous efforts to validate scalp EEG-based source localization. Prior studies have assessed the ability for source localization to predict seizure freedom based on the presence of a single-point solution (dipole or current density maximum) within the TV (17–19), distance from a single-point solution to the TV (8, 20), distance from solution to nearest intracranial electrode involved in seizure onset (21), and sublobar concordance of source localization results and intracranial EEG (22). Meta-analysis of scalp EEG-based source localization studies found 74 and 75% accuracy for interictal and ictal analysis, respectively (23), very similar to the 73% accuracy observed in the present study.

Validation of SEEG-based source localization presented multiple unique challenges. Intracranial data could not be considered ground truth as was done in previous studies. Sublobar localization is insufficient at the SEEG stage, where gyral or even sub-gyral resolution is necessary. This is particularly true for ablative surgical approaches like LITT (highly represented in this cohort) that can target a limited volume of brain. Unlike ablation, resection often extends beyond the core of the epileptogenic zone to include areas involved in early propagation, likely leading to higher rates of seizure freedom vs. LITT (24, 25). For the smaller TV created by ablation, modest errors in localization are conceptually more likely to result in sources outside the TV. A major benefit of this ablation-heavy cohort is that, given the volume limitation of ablation, the TV of seizure-free patients presumably tightly conforms to the epileptogenic zone.

Several strategies were employed to overcome these challenges. A high density of source locations was used, with 2 mm spacing rather than a more conventional spacing of around 5 mm (26), at the cost of increased computational demand (3). Instead of using only single-point solutions (e.g., dipoles, which may be overly simplistic and did not predict seizure outcome in this study), the percentage of overlap between the current density map and the TV was studied. To weigh the results by current magnitude at each location, a model yielding actual currents (SWARM) rather than statistical values (sLORETA) was used, so that the current magnitudes could be added together; this approach may have allowed SWARM rather than sLORETA to predict seizure outcome. Despite these measures, SEEG-based source localization presented significant barriers to validation, particularly in an ablation-heavy cohort. It is thus unsurprising that the accuracy of this method in this cohort did not surpass that of scalp methods.

### Prior Simulation Studies

Few publications describe use of SEEG for source localization, and nearly all data come from computational simulations. The Lorraine group found that simulated dipoles within 3 cm of the nearest contact could be localized within 1 cm under ideal noise conditions (5). Performance was poorer with greater noise, sources not surrounded by contacts, sources near the skull (4), fewer averaged IEDs, and fewer contacts (15). Limiting the solution space to gray matter improved localization accuracy (13), leading to the use of a gray matter grid of solution points in the present study.

Head model complexity for source localization is controversial (3). The Lorraine group observed reasonable performance with a one-sphere model while acknowledging lower accuracy in the frontal and occipital polar regions where the spherical approximation of the cortex is less accurate (5). Superior accuracy has been reported for complex models that incorporate tissue anisotropy and CSF (14). One might therefore suspect less accurate results in patients with prior surgery resulting in CSF-filled cavities, but no effect of prior surgery has been observed in practice (27). In the present study, a three-compartment finite element model was used due to software constraints and served as a middle ground with respect to complexity.

The effects of adding scalp EEG to SEEG have also been simulated (6). Hosseini and colleagues modeled cortical IEDs and used sLORETA to solve the inverse problem. Sources within 1.5 cm of the nearest contact could be localized within 1 cm. Addition of high-density scalp EEG markedly reduced localization error for sources within 3 cm of the nearest depth electrode contact. In contrast, adding low-density scalp EEG in the present study abolished the ability of source localization to discriminate between seizure-free and non-seizure-free patients. High-density EEG is impractical in the presence of depth electrodes, and low-density source localization is known to be less accurate (17). Perhaps most important is that while Hosseini et al. simulated IEDs from random cortical locations, most patients in the present study had deep-seated epileptogenic zones (e.g., mesial temporal lobe). Discharges located far from the brain convexity were frequently not visible on scalp EEG, and addition of scalp recordings decreased the overall SNR. Furthermore, the scalp EEG is often contaminated by discharges from cortical sites driven through neuronal propagation from deep source, and the cortical discharges swamp the smaller volume-conducted driving signals from deep sources (28). In seizure-free patients with a clearly defined epileptogenic zone, the effect of adding scalp noise was to pull the modeled source toward the scalp. A favorable effect of scalp EEG might be seen in a cohort with higher representation of superficial epileptogenic zones.

### Source Localization of Real SEEG

There are few examples of source localization using non-simulated SEEG. The Lorraine group analyzed intracranial stimulation as well as IEDs from a single patient and found results consistent with the simulations discussed previously (4). Yvert et al. used SEEG and a current density method to reconstruct auditory evoked potentials in the supratemporal plane (29). Woolfe et al. developed an automated ictal source localization method based on multi-band fast activity and low-frequency suppression (7). This method was able to identify contacts within the epileptogenic zone with 95% accuracy, based on clinical interpretation of the same SEEG data from patients who became seizure-free.

It bears mention that several studies have validated source localization based on subdural electrodes (30–32). The source-electrode relationship differs between subdural electrodes which sample the cortical surface and depth electrodes which sample the cerebral volume. Because subdural electrodes are spatially intermediate between scalp and depth electrodes, subdural recordings were omitted in the two patients in this cohort who underwent placement of both depth electrodes and subdural strip electrodes.

### Prediction of Far-Field Activity

A major goal of this study was to attempt to localize the epileptogenic zone in the absence of direct (near-field) recordings, in order to circumvent the streetlight effect with an incorrect pre-implantation hypothesis. When contacts within the TV were excluded from analysis, percent overlap between the modeled IZ and the TV did not predict seizure outcome. Excluding contacts in the TV decreased the proportion of interictal current in the TV in seizure-free patients and smeared the current distribution in the overall cohort.

The number of contacts with clear IEDs (i.e., high SNR) was typically small and similar to the number of excluded contacts within the TV. IED selection was not repeated for far-field analysis and may have been difficult in some patients without prominent IEDs outside the TV. Source localization from far-field data is limited by low signal amplitudes and shallow voltage gradients, resulting in low SNR. Central to this issue is the range of the local field potential (LFP) that constitutes SEEG. Research combining multiunit activity and LFP has shown that the LFP is a mixture of local potentials and volume conduction (33). The simulation studies provide some guidance, indicating that subcentimeter localization error is possible with sources within 1.5–3 cm of the nearest contact. In some cases, exclusion of contacts within the TV may leave no channels in which volume conducted far-field signal makes up a significant portion of the LFP, particularly in the presence of near-field noise.

### Limitations and Strengths

The retrospective nature of this study and certain features of the cohort limit generalizability. Source localization was performed retrospectively, and these data provide no direct evidence as to how seizure outcomes would have differed had source localization been incorporated into surgical planning. The 42% rate of seizure freedom at last follow-up is relatively low which may reflect frequent use of LITT as well as high numbers of non-lesional, multi-lesional, and previously operated cases. The number of depth electrodes implanted varies substantially between surgeons, with the average in this cohort (6 electrodes, 46 contacts) on the low end and the French school (well-represented in the source localization literature) on the high end (34). Greater source localization accuracy would be expected from more extensive implantations based upon the Lorraine group's findings. Removing the software-imposed need for common average re-referencing prior to source localization might also improve localization accuracy (5).

Strengths of this study include a minority of cases with a single radiographic lesion (in which the expected contribution of source localization is lower) and the presumed close correspondence between ablation volume and epileptogenic zone previously discussed. The sample size of 26 patients was typical for surgical source localization studies (8, 21, 35). The use of commercially available source localization software may facilitate the replication of these results and the clinical application of this approach.

### Future Directions

Application of this method to different cohorts with more electrodes, more cortical sources, and higher rates of resection vs. ablation could yield different results and further refine invasive and concurrent non-invasive monitoring strategies. Extension of this method to ictal data may more clearly define the epileptogenic zone as suggested by a similar analysis (7), particularly in the absence of the ictal myoelectric artifact seen on scalp EEG.

## Conclusions

SEEG-based interictal source localization accurately represented interictal discharges from SEEG when compared to conventional EEG interpretation. The degree of overlap of SWARM-modeled current density with the TV predicted seizure outcome, thereby validating this technique in corroborating the epileptogenic zone. Source localization of intracerebral recordings is a feasible method for interpreting SEEG in three dimensions, as the prefix “stereo” implies it is meant to be. Seizure outcome could only be predicted in the presence of direct recordings from the surgical target, which may be due to the intrinsic IED field size. Refinement of SEEG-based source localization may yet provide solutions to the streetlight effect.

## Data Availability Statement

The raw data supporting the conclusions of this article will be made available by the authors, without undue reservation.

## Ethics Statement

The studies involving human participants were reviewed and approved by University of Chicago Institutional Review Board. Written informed consent from the participants' legal guardian/next of kin was not required to participate in this study in accordance with the national legislation and the institutional requirements.

## Author Contributions

DS: conceptualization, methodology, investigation, formal analysis, data curation, writing-original draft, and writing-review and editing. YE: investigation and writing-review and editing. PW: conceptualization, writing-review and editing, and supervision. NI: conceptualization, resources, writing–review and editing. DN: conceptualization, investigation, resources, writing–review and editing, and supervision.

## Conflict of Interest

The authors declare that the research was conducted in the absence of any commercial or financial relationships that could be construed as a potential conflict of interest.

## Publisher's Note

All claims expressed in this article are solely those of the authors and do not necessarily represent those of their affiliated organizations, or those of the publisher, the editors and the reviewers. Any product that may be evaluated in this article, or claim that may be made by its manufacturer, is not guaranteed or endorsed by the publisher.

## Abbreviations

IZ: irritative zone
LITT: laser interstitial thermal therapy
SEEG: stereo-electroencephalography
sLORETA: standardized low resolution tomographic analysis
SWARM: sLORETA-weighted accurate minimum norm
TV: treatment volume.
